# Life Table Parameters and Digestive Enzyme Activity of *Spodoptera littoralis* (Boisd) (Lepidoptera: Noctuidae) on Selected Legume Cultivars

**DOI:** 10.3390/insects13070661

**Published:** 2022-07-21

**Authors:** Seyed Ali Hemmati, Parviz Shishehbor, Lukasz L. Stelinski

**Affiliations:** 1 Department of Plant Protection, Faculty of Agriculture, Shahid Chamran University of Ahvaz, Ahvaz 61357-43311, Iran; pshishehbor@scu.ac.ir; 2 Department of Entomology and Nematology, Citrus Research and Education Center, University of Florida, Lake Alfred, FL 33850, USA; stelinski@ufl.edu

**Keywords:** Egyptian cotton leafworm, reproductive potential, enzyme activity, host plant resistance

## Abstract

**Simple Summary:**

*Spodoptera littoralis* (Boisd) is a highly polyphagous and destructive pest, which attacks a wide range of economically important crops throughout the world. The extensive use of conventional insecticides for management of *S.* *littoralis* has resulted in development of resistance to major classes of pesticides and can have a negative impact on the environment. It is necessary to investigate alternative pest management approaches that are more cost-effective and sustainable than conventional insecticides. The aim of this study was to identify potential sources of variation in resistance/susceptibility of legume cultivars to *S.* *littoralis*, and to describe potential interactions between cultivar traits and digestive function of this pest. We conducted life table analyses and assessed both proteolytic and amylolytic activities of *S.* *littoralis* on 11 common legume cultivars. The findings indicated that *S. littoralis* reared on the common bean, Arabi, displayed the highest intrinsic rate of increase, while the lowest was recorded on the cowpea, Mashhad. Developmental time of *S. littoralis* larvae was negatively correlated with protein content, while amylolytic activity was positively correlated with starch content of legumes. Our results revealed that the Mashhad cultivar exhibited tolerance traits against *S.* *littoralis*, which may prove useful for integrated programs that aim to reduce chemical inputs.

**Abstract:**

*Spodoptera littoralis* (Boisd) is a highly destructive pest that attacks a large number of economically important crops. We examined life table parameters as well as activity of major digestive enzymes of *S. littoralis* larvae in response to protein and starch contents across 11 legume cultivars to identify potential resistance traits. The results showed that *S. littoralis* reared on the common bean, Arabi, displayed the highest intrinsic rate of increase (*r*), while the lowest was recorded on the cowpea, Mashhad. Also, the highest net reproductive rate (*R*_0_) was obtained in those insects reared on the Arabi cultivar. Larvae displayed the highest and lowest proteolytic activities when fed on Mashhad and Arabi cultivars, respectively. The highest amylolytic activity was quantified in larvae that fed on the Arabi and 1057 cultivars, while the lowest occurred in larvae feeding on Yaghout and Mashhad cultivars. Developmental time of *S. littoralis* larvae was negatively correlated with protein content, while amylolytic activity was positively correlated with starch content of legumes. Our findings indicate that Arabi was a susceptible cultivar, while Mashhad exhibited tolerance traits against *S. littoralis*. These results should facilitate selection of legume cultivars for production or breeding efforts that involve *S. littoralis* management.

## 1. Introduction

The Egyptian cotton leafworm, *Spodoptera littoralis* (Boisd) (Lepidoptera: Noctuidae), is a highly polyphagous and destructive pest which attacks a wide range of economically important crops throughout the world [1,2,3,4]. Lepidopteran larvae are of global phytosanitary concern due to their ability to attack a variety of plant structures, negatively affecting crop quality and causing economic losses [5]. The extensive use of conventional insecticides for management of *S. littoralis* has resulted in development of resistance to major classes of pesticides [6], and can have a negative impact on the environment [7]. Given the emergence of resistance and environmental hazards, it is necessary to investigate alternative pest management approaches that are more cost-effective and sustainable than conventional insecticides [8].

The use of resistant plant cultivars is an eco-friendly and effective approach within a framework of integrated pest management [9,10]. Plant cultivars can be identified or bred for resistance or tolerance traits that can minimize the survival, larval growth, longevity, reproductive potential, and population growth of herbivorous insects [11,12,13]. Selection of cultivars that display resistance or tolerance traits for pest management requires an understanding of pest performance and life cycle parameters on those selections [14,15,16].

Herbivore performance and population growth is affected by the nutritional content and biochemical attributes of host plants [17,18,19]. Therefore, assessment of the biochemical metabolites characterizing potential selections for cultivation may help explain differences in demographic and physiological responses of herbivores and assist in selection of resistance traits [13,20]. For example, elucidating digestive physiology of pests has benefited development of target-specific insecticides [21,22,23]. Highly polyphagous herbivores, such as *S.* *littoralis*, have sophisticated mechanism(s) to regulate their digestive proteases [24]; midgut proteases as well as α-amylases are two groups that may prove particularly important for digestion of legumes [25].

There is limited published information on comparative performance and digestive responses of *S. littoralis* among possible cultivatable hosts [26,27,28], and in particular no comparative information from legumes, which are the main hosts of *S. littoralis*. Nutritional performance of *S. littoralis* on bean cultivars, including red kidney bean, common bean, white kidney bean and cowpea was demonstrated by Shishehbor and Hemmati (2022), who reported that the common bean, Arabi, and white kidney bean, Almas, were suitable hosts for *S. littoralis*. However, the cowpea, Mashhad, was identified as unsuitable [4].

In the current investigation, we conducted life table analyses and assessed both proteolytic and amylolytic activities of *S.* *littoralis* on 11 common legume cultivars. Our goals were to identify potential sources of variation in resistance/susceptibility of legume cultivars to this damaging pest, and to describe potential interactions between cultivar traits and digestive function of this pest. Our results may improve selection of cultivars that exhibit tolerance traits against *S.* *littoralis* for use in integrated programs that aim to reduce chemical inputs.

## 2. Materials and Methods

### 2.1. Legume Cultivars

Seeds of 11 leguminose cultivars including *Phaseolus vulgaris* L. (red kidney bean; Dadfar, Ofogh, and Yaghout cultivars), common bean (Arabi, Ghaffar, Sadri, and Saleh cultivars), white kidney bean (Almas and Dorsa cultivars) and *Vigna sinensis* L. (cowpea; Mashhad and 1057 cultivars) were obtained from the Seed and Plant Research Improvement Institute (Karaj, Iran). Legume seeds were cultivated in plastic pots in the greenhouse facility of Shahid Chamran University of Ahvaz, located in Ahvaz, Iran. Leaves were removed from plants prior to assay and transferred to the growth chamber (25 ± 1 °C, 65 ± 5% RH, and a 16:8 h light:dark photoperiod) as described below for larval feeding.

### 2.2. Insect Rearing

The *S.* *littoralis* culture was initiated from bean fields in the Karun region, Khuzestan province, of Iran. The larvae were reared on leaves of various legumes in the growth chamber under conditions given above. Before experiments were initiated, larvae were reared on leaves of each cultivar stated above for two generations. Then, the third-generation colony was used for quantifying life table parameters on each cultivar evaluated and for digestive enzyme quantifications. This was done to minimize the possible effects of host rearing/experience on comparisons of cultivars as host plants.

### 2.3. Life History Parameters and Body Mass in Immature Stages of S. littoralis on Legume Cultivars

Life table parameters of *S. littoralis* were initiated by obtaining eggs of standardized age that were all laid within a 12 h period on each cultivar. We established a total of 50 eggs laid per plant at the onset of the experiment, which was achieved by carefully removing additional eggs with a fine brush. After hatching, neonate larvae were individually transferred into Petri dishes (diameter 9 cm, depth 2 cm) on the same leaves. The duration prior to hatching (egg period), larval, pre-pupal, and pupal incubation period were subsequently quantified. Furthermore, we determined the survival rate until adult emergence. Petri dishes were maintained in the growth chamber (25 ± 1 °C, 65 ± 5% RH, and a 16:8 h light:dark photoperiod) and checked daily to record survivorship and period of developmental stages on each cultivar. As larvae matured, fresh leaves from the original plants were replaced every 24 h for each cultivar. Additionally, a strip of wet cotton was wrapped around the petiole of each leaf to delay desiccation. The pre-pupal and pupal weights of *S. littoralis* were estimated based on dry weight 24 h after their emergence and pupation on each cultivar. Leaves from 25 plants were evaluated per cultivar.

### 2.4. Life Table Variables of Adult S. littoralis Developing on Various Legume Cultivars

After emergence of adult female and male individuals, each pair of moths from the same legume cultivar were paired into plastic tubes fitted with mesh lids containing leaves of the same legume cultivar as laying egg substrates. A source of carbohydrate was provided in the egg-laying container assays with a small cotton ball dipped in honey solution (10%). The number of eggs laid by each adult female was counted per 24 h. Eggs laid by each pair were collected daily and replaced with fresh leaves, and the process was continued until all adults died. Longevities of female and male adults, fecundity (number of eggs laid during reproductive time), oviposition period, adult pre-ovipositional period (APOP), and total pre-ovipositional period (TPOP) were quantified for each of the 11 legume cultivars.

### 2.5. Life Table Analysis

Data analyses were based on the age-stage, two-sex life table method [29,30,31].

The age-specific survivorship (*l_x_*) was estimated as:

$$l_{x}=\overset{n}{\underset{j=1}{\sum }}s_{xj}$$

where, *x* is the age, *j* is the stage, and *n* is the number of stages.

The age-specific fecundity (*m_x_*) was calculated via the formula:

$$m_{x}=\overset{n}{\underset{j=1}{\sum }}s_{xj}f_{xj}/\overset{n}{\underset{j=1}{\sum }}s_{xj}$$

The net reproductive rate (*R*_0_) was also estimated as:

(1) $$R_{0}=\overset{\infty }{\underset{x=0}{\sum }}l_{x}m_{x}$$

The *l_x_* and *m_x_* values were used to calculate the intrinsic rate of increase (*r*) from the following formula:

(2) $$\overset{\infty }{\underset{x=0}{\sum }}e^{-r\;\left(x+1\right)}\;l_{x}m_{x}=1$$

Furthermore, the finite rate of increase (*λ*), and mean generation time (*T*) parameters were calculated with the equations: *λ* = *e^r^*, and *T* = (ln *R*_0_)/*r*, respectively.

### 2.6. Preparation of Midgut Extracts

After 24 h of feeding on each of the legume cultivars tested, larvae (sixth instar) were incapacitated by chilling on ice, and quickly dissected under a stereomicroscope. There were 20 larvae collected at random from 20 plants per cultivar. The haemolymph was washed away with precooled distilled water and unwanted tissues were removed, and the midguts were transferred to distilled water and homogenized in a glass homogenizer on ice. Next, the homogenates were centrifuged at 14,000× *g* for 10 min at 4 °C, and the clear supernatants were aspirated and kept at −20 °C until enzyme assays were performed.

### 2.7. Determination of Proteolytic Activity of S. littoralis after Feeding on Various Legume Cultivars

General proteolytic activity of midgut extracts was determined using a 2% (*w*/*v*) solution of azocasein as a substrate in the universal buffer system (50 mM sodium phosphate-borate) over a pH range of 7–12. The reaction mixture containing 50 μL of the midgut extract and 80 μL of the substrate in 50 mM universal buffer was incubated at 37 °C for 50 min. Proteolysis was stopped by adding 100 µL of 30% trichloroacetic acid (TCA), followed by cooling at 4 °C for 30 min and centrifugation at 14,000× *g* for 10 min. An equal volume of 2 M NaOH was added to the supernatant, and the absorbance was measured at 440 nm. One unit of proteolytic activity was defined as the quantity of enzyme (mg) that produces an increase in the optical density by 0.1 per minute in 1 mL of the reaction mixture under the assay conditions [32]. All experiments were performed in four replicates using appropriate controls. Furthermore, protein concentrations were determined using the Bradford (1976) protein assay [33], and known amounts of bovine serum albumin (BSA) (2, 1.5, 1, 0.5, 0.25, 0.125, and 0.063 mg mL^−^^1^) were used to generate a standard curve.

### 2.8. Determination of Amylolytic Activity of S. littoralis on Legume Cultivars

Digestive amylolytic activity of midgut extracts was assessed utilizing the dinitrosalicylic acid (DNS) procedure, with 1% starch as a substrate in the universal buffer system (10 mM succinate-glycine-2, morpholinoethan sulfunic acid) over a pH range of 5–12. The mixture containing midgut extracts and 1% starch was incubated at 37 °C for 30 min. The reaction was then stopped by adding 50 μL of DNS and heating in boiling water for 10 min [34]. The absorbance was recorded at 540 nm after cooling the mixture on ice. The amount of enzyme needed to generate one mg of maltose in 30 min at 37 °C was considered a unit of amylase activity under the assay conditions. Assays were carried out in four replicates using appropriate controls.

### 2.9. Biochemical Characteristics of Legume Cultivars

Biochemical properties of various legume cultivars, including protein and starch contents were assessed. Each legume cultivar was analyzed using four replicate plants. Leaves were used to measure all phytochemicals.

The Bradford method was used to estimate the protein content in leaves. Briefly, 200 mg of the powdered leaves of each cultivar was homogenized in 10 mL of distilled water, and then 100 µL of the homogenate was mixed to 3 mL of Bradford reagent. The samples absorption was read at 595 nm using bovine serum albumin as a standard [33].

The method of Bernfeld (1955) was used to measure the starch content of leaves [34]. A quantity of the powdered leaves (200 mg) of each legume cultivar was homogenized in 35 mL of distilled water and heated to boiling. Then, 100 µL of each sample was added with 2.5 mL of iodine reagent (0.2% KI and 0.02% I_2_), and the absorption was recorded at 580 nm.

### 2.10. Statistical and Cluster Analysis

Enzyme activity and insect weight data were first tested for normality (Shapiro–Wilk coefficient > 0.05), and subsequently analyzed using one-way analysis of variance (ANOVA) according to a completely randomized design. Comparison of means was carried out using Turkey’s *post hoc* Honestly Significant Difference (HSD) test with an alpha cut-off of 1% (α= 0.01) by the statistical software SPSS v. 22.0. The variances of population growth parameters were calculated via the bootstrap method and the bootstrap values were compared by paired bootstrap tests [35]. Furthermore, a cluster analysis was carried out to find groups of legume cultivars with similar traits using the life table parameters and enzyme activity levels from *S. littoralis* larvae as variables. We determined their contribution to performance of this pest on legumes by a two-step cluster approach using Ward’s minimum-variance hierarchical clustering method in SPSS 22.0 statistical software. Pearson’s correlation analysis was used to determine if there were relationships between life table parameters or digestive enzyme activity of *S. littoralis* and biochemical traits of the legume cultivars tested [36].

## 3. Results

### 3.1. Life History Parameters and Body Weight

Durations of immature stages and survival of *S. littoralis* on various legume cultivars are presented in Table 1. Durations of egg, larval, pre-pupal, and pupal periods, as well as developmental time of *S. littoralis* varied significantly among the legume cultivars (*p* < 0.01). The highest and lowest egg incubation periods were obtained on Sadri (4.364 day) and Arabi (3.937 day) cultivars, respectively. Larvae fed with Ofogh (21.592 day) and Mashhad (21.758 day) cultivars exhibited the longest larval periods, while those fed with Almas (19.712 day) and Ghaffar (20.043) had the shortest periods. The pre-pupal period was significantly shorter in larvae reared on the common bean, Arabi (1.515 day), than the other cultivars tested (Table 1). The longest pupal period was recorded on red kidney bean, Ofogh (10.840 day), whereas the shortest pupal periods occurred on Arabi (9.793 day) and Almas (9.888 day) cultivars. Furthermore, the longest and shortest developmental times from egg to adult emergence were observed on Mashhad (38.740) and Arabi (32.515 day) cultivars, respectively.

**Figure 1:**
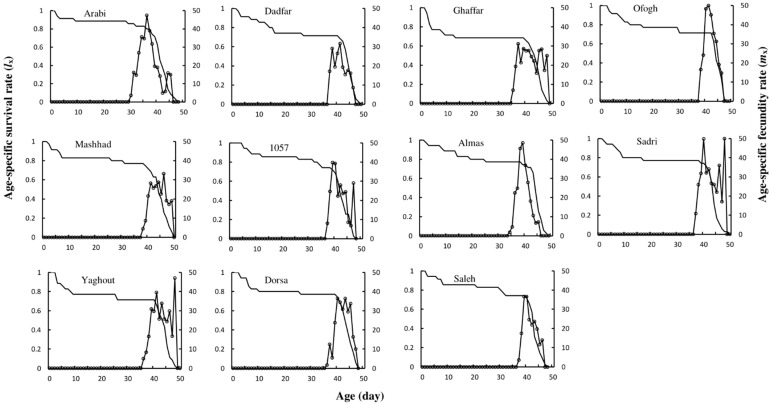
Age-specific survival rate (*l_x_*) and age-specific fecundity (*m_x_*) of *Spodoptera littoralis* on various legume cultivars.

**Figure 2:**
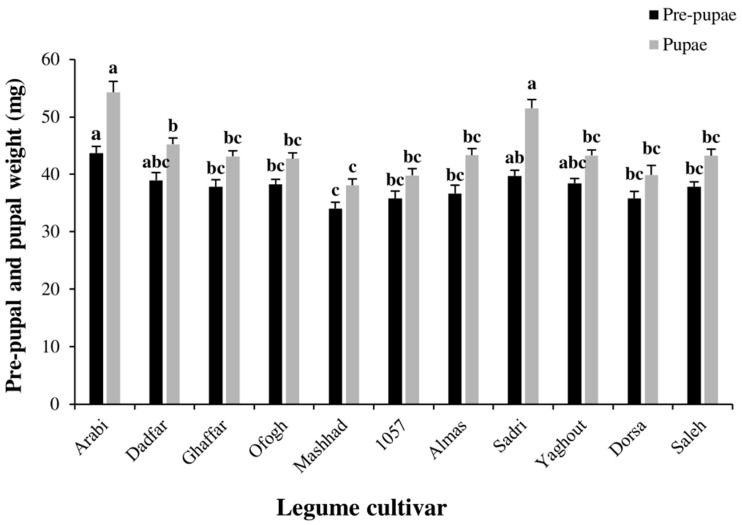
Pre-pupal and pupal weights of *Spodoptera littoralis* reared on various legume cultivars. Each column represents the mean of 25 independent estimations ± standard error (SE). Different letters indicate statistically significant differences (Tukey, *p* < 0.01).

The age-specific survival (*l_x_*) and fecundity (*m_x_*) rates of *S. littoralis* fed on various legume cultivars are plotted in Figure 1. Overall, age-specific survival curves were similar among the tested cultivars, and *S. littoralis* successfully reproduced and developed on each of the cultivars investigated. The greatest and lowest pre-pupal weights (*F*_10, 264_ = 4.76; *p* < 0.01) occurred on the Arabi (43.671 mg) and cowpea Mashhad (34.020 mg), respectively. The greatest and lowest pupal weights (*F*_10, 264_ = 14.589; *p* < 0.01) occurred on Arabi (54.300 mg) and Mashhad (41.820 mg) cultivars, respectively (Figure 2).

### 3.2. Adult Life Table Variables

Adult longevity and reproductive performance of *S. littoralis* reared on the various legume cultivars are presented in Table 2. Female *S. littoralis* reared on the Mashhad (1.643 day) and Arabi (1.053 day) cultivars exhibited the longest and shortest adult pre-oviposition periods (APOP), respectively. The longest total pre-oviposition period (TPOP) occurred on the Ofogh (39.398 day) and Mashhad (39.501 day) cultivars, while the shortest TPOP was documented on the Arabi (33.470 day) cultivar. The longest oviposition period occurred on the common bean, Ghaffar (7.138 day), while the shortest periods were recorded on red kidney bean, Ofogh (5.401 day), and 1057 (5.282 day) cultivars. Total fecundity was highest on Arabi (445.988) and Ofogh (422.150). Maximum adult female longevity occurred on the white kidney bean, Almas (10.001 day), while the lowest value was documented on red kidney bean, Ofogh (7.070 day). Male adult longevity was highest when reared on Arabi (9.303 day) and lowest when reared on the common bean, Sadri (5.099 day) (Table 2).

There were significant differences among all population growth parameters of *S. littoralis* reared on the various legume cultivars tested (Table 3) (*p* < 0.01). The highest net reproductive rate (*R*_0_) value was obtained in those insects reared on Arabi (242.34 offspring). Female *S. littoralis* exhibited the highest and lowest intrinsic rate of increase (*r*) when reared on common bean, Arabi (0.150 day^−1^) and cowpea, Mashhad (0.111 day^−1^), respectively. The differences in the finite rate of increase (*λ*) were similar to the *r* parameter and the *λ* variable was significantly affected by legume cultivar. Adult *S. littoralis* reared on the cowpea, Mashhad (42.60 day), exhibited the longest generation time (*T*), while the shortest occurred on the Arabi cultivar (36.27 day) (Table 3).

### 3.3. The Activity of Digestive Enzymes

**Figure 3:**
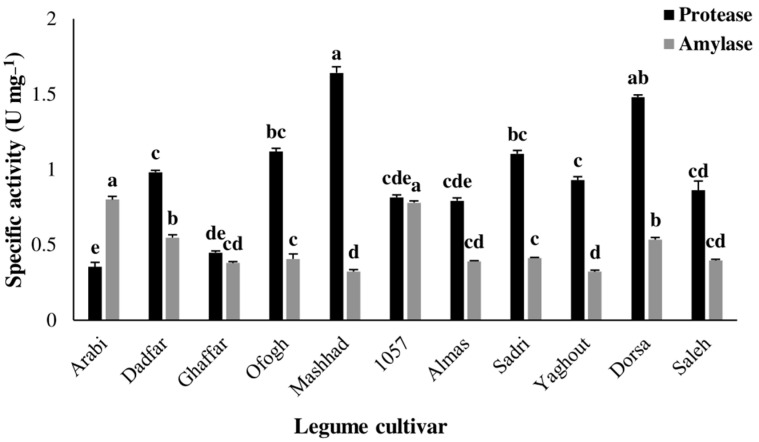
General proteolytic and amylolytic activity of midgut extracts from *Spodoptera littoralis* larvae reared on various legume cultivars. Each column represents the mean of four independent estimations ± standard error (SE). Different letters indicate statistically significant differences (Tukey, *p* < 0.01).

General proteolytic activity (*F*_10, 33_ = 16.52; *p* < 0.01) and amylolytic activity (*F*_10, 33_ = 30.189; *p* < 0.01) in the larval midgut of *S.* *littoralis* reared on various hosts until the sixth instar are shown in Figure 3. The findings indicated that both proteases and amylases were most active in the larval midgut extract under alkaline conditions of pH 11 and 10, respectively. The activities of digestive proteases and amylases in the larval midgut were significantly affected by the legume cultivar consumed by *S.* *littoralis*. Larvae fed on Mashhad (1.642 U mg^−1^) and Arabi (0.353 U mg^−1^) cultivars exhibited the highest and lowest levels of proteolytic activity, respectively. The highest amylolytic activities were detected in larvae reared on the cultivars, Arabi (0.800 U mg^−1^) and 1057 (0.780 U mg^−1^). In contrast, the lowest amylolytic activities were observed in larvae fed on the cultivars, Yaghout (0.321 U mg^−1^) and Mashhad (0.322 U mg^−1^).

### 3.4. Cluster Analysis

**Figure 4:**
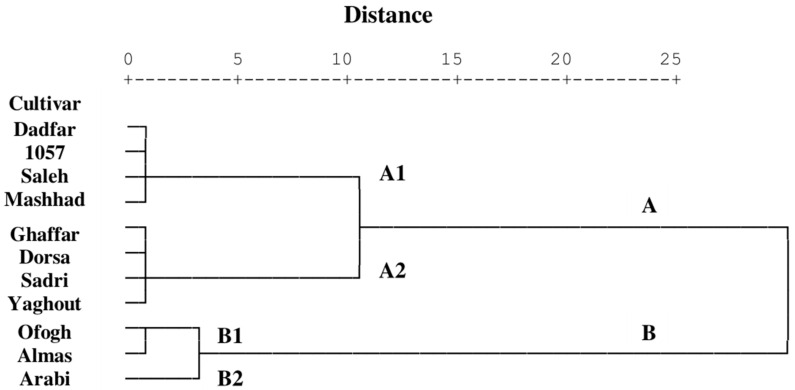
Dendrogram from cluster analysis of various legumes based on life table parameters and digestive proteolytic and amylolytic activities of *Spodoptera littoralis* in response to feeding on various legumes cultivars (Ward’s method).

The dendrogram based on body weight, demographic characteristics as well as proteolytic and amylolytic activities of *S.* *littoralis* revealed two groups designated A and B (Figure 4). Cluster A consisted of two sub-clusters: A_1_ (Dadfar, 1057, Saleh, and Mashhad cultivars), which shared characteristics distinguishing them as the most resistant hosts, and A_2_ (Ghaffar, Dorsa, Sadri, and Yaghout cultivars), which were designated as relatively unsuitable hosts for *S.* *littoralis*. However, sub-cluster B_1_ contained Ofogh and Almas cultivars, which were considered relatively suitable hosts for *S.* *littoralis.* The results also delineated a sub-cluster B_2,_ which consisted of common bean, Arabi, and which was the most susceptible cultivar for *S.* *littoralis*.

### 3.5. Biochemical Characteristics of Various Legume Cultivars

The primary legume metabolites quantified from the cultivars analyzed are given in Table 4. There were significant differences among the metabolites and the highest protein content was measured from the white kidney bean, Almas (0.343 mg/mL); whereas the lowest protein content was quantified from the red kidney bean, Dadfar (0.223 mg/mL) (*F*_10, 22_ = 25.28; *p* < 0.01). The highest starch content was measured in common bean, Arabi (2.569 mg/mL), while the lowest starch content occurred in the red kidney bean, Yaghout (2.130 mg/mL) (*F*_10, 22_ = 5.829; *p* < 0.01).

### 3.6. Correlation Analysis

The correlation analysis between life table parameters and physiological traits of *S. littoralis* with primary metabolites quantified from the various cultivars tested are given in Table 5. Developmental time of *S. littoralis* larvae was significantly and negatively correlated (*r* = −0.454) with protein content of legumes (*p* < 0.01). In contrast, there was no significant correlation between pupal weight, fecundity, *R*_0_ and *r* traits of *S.* *littoralis* with the protein content of legumes (*p* > 0.05). Likewise, amylolytic and proteolytic activities in larval midguts were not significantly correlated with protein content (*p* > 0.05). The *r* parameter of *S. littoralis* was positively correlated with starch content of legume cultivars (*r* = 0.609) (*p* < 0.01). There was a significant positive correlation between starch content of various legumes and amylolytic activity in larvae (*r* = 0.619) (*p* < 0.01). Conversely, the pupal weight, development time, fecundity, *R*_0_ and proteolytic of activity of *S. littoralis* were not significantly correlated with starch content of the various legumes tested (*p* > 0.05) (Table 5).

## 4. Discussion

The Egyptian cotton leafworm is one of the most destructive and highly polyphagous pests worldwide, infesting a broad range of crops and vegetables [26,37]. Our results indicate that performance and population growth parameters of *S. littoralis* were significantly affected by cultivar among a variety of legumes commonly cultivated for human consumption. The quality of plant cultivars can play a major role in the population dynamics of pests by influencing developmental time and reproductive performance [19]. In particular, highly suitable hosts are associated with large population growth in *S. littoralis* [38]. Despite the economic importance of *S. littoralis* on legumes, there have been no life table or performance investigations published for this pest on this important crop. Previous studies with various other plant species have revealed significant variation in host plant suitability to *S. littoralis* [4,26,27,28]. In general, the use of plant tolerance or resistance traits as a means of managing *S. littoralis* has been relatively neglected.

The life table analyses revealed that *S. littoralis* successfully develops from egg to adult on all of the tested legume cultivars. However, there were apparent differences in the nutritional potential and growth rates of *S. littoralis* among various legume cultivars. The red kidney bean, Ofogh, and the cowpea, Mashhad, appeared least suitable as sources of nutrition and supported the lowest growth rate of developing larvae. Variation in the period from egg to adult emergence of *S. littoralis* among the various cultivars tested may also be related to differences in macronutrients and biochemical characteristics between the legumes investigated. The shortest developmental time occurred on the common bean, Arabi, and may be related to the high starch content characterizing this cultivar. Furthermore, secondary plant metabolites could also be factors affecting performance and development of *S. littoralis* [4]. Adult reproductive potential depends fully on nutrition acquired during the immature stage and therefore duration of immature stages affects the fecundity and longevity of adults [16]. Accordingly, there were significant differences in life table parameters of *S. littoralis* on the 11 cultivars evaluated; Arabi and Mashhad cultivars supported the highest and lowest reproductive potential of adults, respectively.

There were significant correlations between growth and reproduction potential of *S. littoralis* and primary plant metabolites quantified among hosts, including protein and starch contents. The duration of immature stages of *S. littoralis* feeding on legumes was negatively correlated with levels of macronutrients, particularly protein. The growth rate of *S. littoralis* on the Arabi cultivar in particular may be explained by the relatively high starch and protein contents characterizing this cultivar, which was associated with corresponding high body mass and pupal weights of *S. littoralis* developing on this host. In addition, the intrinsic rate of increase (*r*) of *S. littoralis* was positively correlated with starch content, which is congruent with the relatively high *r* value characterizing *S. littoralis* that developed on Arabi. Furthermore, these results are consistent with the previous investigations that related macronutrient and biochemical traits of various plants to herbivore performance [4,18,39,40]. The *r* value is related to life history traits, such as developmental time, survival rate, and fecundity. Therefore, *r* can be a useful indicator of the degree of susceptibility (or resistance) of plants to herbivores [31,41].

We also measured protease and amylase activities in the midgut of *S.* *littoralis* that fed on various legume cultivars. In general, plants protect themselves against herbivores by producing various digestion inhibitors, restricting nutrient uptake. To compensate, insect herbivores increase production of digestive enzymes or upregulate production of inhibitor-insensitive enzymes in the midgut [42,43,44]. Plant protease inhibitors (PIs) are an essential group of defensive compounds commonly found in plant storage organs such as seeds and tubers, which limit assimilation of dietary proteins [22,45]. In addition to protease inhibitors, amylase inhibitors are commonly found in plants, and their inhibitory effect on the activity of digestive amylases in insects reduces digestibility of starchy compounds [46,47,48,49]. Previous studies have revealed that plant produced inhibitory compounds selectively act against insect amylases and proteases, and this specificity is essential for protecting endogenous plant amylases [50,51]. The pH and food content in the midgut are two main factors inherent to herbivores that can change the activity of digestive enzymes [52]. Regardless of the legume cultivar tested as a host in this study, midgut extracts from *S.* *littoralis* larvae indicated that optimal proteolytic and amylolytic activities occurred at a pH of 11 and 10, respectively.

Polyphagous insects can rapidly modify their digestive enzyme profiles in response to ingested PIs by up- and down-regulation of gut proteases [42,51]. As a highly polyphagous pest, *S.* *littoralis* is expected to demonstrate diverse and flexible responses in gut protease secretion in response to variation in host quality. Our results revealed that both proteolytic and amylolytic activities measured under optimal pH conditions differed widely among *S.* *littoralis* larvae reared on different legume cultivars. Among the tested legume cultivars, the highest general proteolytic activity was observed in larvae reared on the Mashhad cultivar. This high protease activity could be a response to the high protein content characterizing this cultivar or an adaptative response to ingestion of potent PIs produced by the Mashhad cultivar. These hypotheses require further investigation.

Shishehbor and Hemmati (2022) demonstrated that *S.* *littoralis* feeding on the Mashhad cultivar exhibited relatively low efficiencies of conversion of ingested (EDI) and digested (ECD) food [4], which are known to be related to activity of digestive enzymes [53]. Therefore, low values of ECI and ECD of *S.* *littoralis* larvae reared on the Mashhad cultivar may be associated with nutritional deficiencies or because of secondary metabolites characteristic of this host.

A cluster analysis based on the life table analysis as well as proteolytic and amylolytic activities was expected to differentiate among cultivars with similar characteristics based on their suitability to *S.* *littoralis*. Therefore, these cultivar groupings may reflect similarities in biochemical properties among cultivars. The cultivars in sub-cluster B_1_ (Ofogh and Almas) and sub-cluster B_2_ (Common bean Arabi) were characterized as relatively suitable or susceptible to *S.* *littoralis*, respectively. However, those cultivars which were grouped into sub-clusters A_1_ (Dadfar, 1057, Saleh and Mashhad) and A_2_ (Ghaffar, Dorsa, Sadri and Yaghout) exhibited characteristics that would classify them as relatively resistant or unsuitable legumes for *S.* *littoralis*, respectively. Interestingly, the unsuitability of the Mashhad cultivar, demonstrated by poor performance as measured by life table parameters, and pupal weight, correlated with the highest general proteolytic activity observed in larvae reared on this cultivar.

Collectively, the current results and those of Shishehbor and Hemmati (2022) indicate that the cowpea, Mashhad, is a poor host for *S.* *littoralis* [4]. These investigations suggest that the Mashhad cultivar may be characterized by PIs that contribute to the poor performance of *S.* *littoralis* reared on this cultivar. Further research is needed to identify potential specific compound(s) associated with this cultivar that may delay growth and development of *S. littoralis*. Moreover, a wider range of cultivars remains to be tested to allow selection of cultivars that exhibit a combination of optimal tolerance traits against *S. littoralis*, without negatively affecting traits required for optimal cultivation and human consumption. Comprehensive molecular and biochemical analysis of midgut proteases and carbohydrates may shed light on the adaptive responses of larvae feeding on different cultivars.

## 5. Conclusions

Our results indicate that the Arabi cultivar was the most suitable host for population growth of *S. littoralis* (high *R*_0_, *r*, and *λ*). Interestingly, the Arabi cultivar is native to the Khuzestan province region of Iran and therefore may indicate potential adaptations between the pest and this cultivar, given the long period of potential co-evolution. In contrast, *S. littoralis* performed worst on the Mashhad cultivar, which may be associated with tolerance traits, such as high concentrations of digestion inhibitors or secondary metabolites and/or sub-optimal levels of primary metabolites. Furthermore, our results revealed considerable variation in primary metabolite contents, including protein and starch, among the legume cultivars investigated, which may be associated with differences in reproductive and population growth potential quantified in *S. littoralis* [54,55]. Overall, our results suggest that the Mashhad cultivar may be a useful selection for cultivation in areas where *S. littoralis* occur in high population densities and where access to other tools, such as insecticides, may be more limited.

## Figures and Tables

**Table 1:** The duration (day) and survival of immature stages (mean ± SE) of *Spodoptera littoralis* reared on various legume cultivars.

Legume Cultivar	Egg Incubation	Larval Period	Pre-Pupal Period	Pupal Period	Development Time
Arabi	3.937 ± 0.043 c	17.387 ± 0.305 d	1.515 ± 0.102 c	9.793 ± 0.157 e	32.515 ± 0.393 d
Dadfar	4.095 ± 0.051 b	20.770 ± 0.157 b	1.960 ± 0.040 a	10.240 ± 0.103 d	37.041 ± 0.251 bc
Ghaffar	4.221 ± 0.079 ab	20.043 ± 0.200 c	1.542 ± 0.103 bc	10.293 ± 0.140 cd	36.086 ± 0.336 c
Ofogh	4.218 ± 0.072 ab	21.592 ± 0.255 a	1.999 ± 0.017 a	10.840 ± 0.167 a	38.639 ± 0.317 a
Mashhad	4.188 ± 0.69 ab	21.758 ± 0.404 a	2.283 ± 0.280 a	10.41 ± 0.347 abcd	38.740 ± 0.473 a
1057	4.258 ± 0.073 ab	20.770 ± 0.200 b	1.787 ± 0.077 ab	10.384 ± 0.144 bcd	37.083 ± 0.251 b
Almas	4.121 ± 0.057 b	19.712 ± 0.142 c	2.001 ± 0.074 a	9.888 ± 0.080 e	35.702 ± 0.180 c
Sadri	4.364 ± 0.082 a	20.852 ± 0.187 b	1.814 ± 0.075 a	10.630 ± 0.157 abc	37.668 ± 0.250 b
Yaghout	4.129 ± 0.060 b	21.006 ± 0.223 ab	1.960 ± 0.040 a	10.763 ± 0.154 ab	37.849 ± 0.359 ab
Dorsa	4.212 ± 0.071 ab	20.786 ± 0.180 b	1.926 ± 0.050 a	10.555 ± 0.133 abcd	37.482 ± 0.238 b
Saleh	4.243 ± 0.075 ab	20.655 ± 0.114 b	2.034 ± 0.035 a	10.230 ± 0.099 d	37.115 ± 0.168 b

Means followed by different letters in each column are significantly different (Paired bootstrap test, *p* < 0.01).

**Table 2:** The reproductive period, fecundity, and adult longevity (mean ± SE) of *Spodoptera littoralis* reared on various legume cultivars.

Legume Cultivar	APOP (Day)	TPOP (Day)	Oviposition Period (Day)	Fecundity (Offspring)	Female Adult Longevity (Day)	Male Adult Longevity (Day)
Arabi	1.053 ± 0.052 d	33.470 ± 0.443 e	6.849 ± 0.457 ab	445.988 ± 25.16 a	9.851 ± 0.527 ab	9.303 ± 0.445 a
Dadfar	1.073 ± 0.072 cd	38.643 ± 0.370 ab	5.853 ± 0.354 bc	308.520 ± 25.412 b	8.430 ± 0.364 cd	7.458 ± 0.433 b
Ghaffar	1.469 ± 0.161 ab	37.203 ± 0.425 cd	7.138 ± 0.288 a	363.198 ± 7.046 ab	9.001 ± 0.215 bc	8.216 ± 0.494 ab
Ofogh	1.331 ± 0.125 abc	39.398 ± 0.267 a	5.401 ± 0.232 c	422.150 ± 12.08 a	7.070 ± 0.366 f	6.097 ± 0.402 cd
Mashhad	1.643 ± 0.131 a	39.501 ± 0.460 a	5.92 ± 0.447 bc	289.83 ± 11.51 b	7.351 ± 0.505 def	5.462 ± 0.285 cd
1057	1.214 ± 0.113 bcd	38.145 ± 0.249 bc	5.282 ± 0.467 c	311.258 ± 15.90 b	7.137 ± 0.494 ef	6.079 ± 0.492 cd
Almas	1.251 ± 0.143 bcd	36.871 ± 0.281 d	6.382 ± 0.451 abc	406.011 ± 34.40 ab	10.001 ± 0.396 a	7.991 ± 0.553 ab
Sadri	1.236 ± 0.104 bcd	38.703 ± 0.312 ab	5.825 ± 0.280 bc	345.396 ± 9.11 ab	7.532 ± 0.325 def	5.099 ± 0.375 d
Yaghout	1.307 ± 0.130 abcd	38.701 ± 0.441 ab	5.92 ± 0.135 bc	391.449 ± 10.77 ab	8.001 ± 0.195 de	5.582 ± 0.304 cd
Dorsa	1.425 ± 0.172 abc	38.429 ± 0.355 ab	5.857 ± 0.367 bc	357.850 ± 11.19 ab	7.569 ± 0.443 def	6.157 ± 0.316 c
Saleh	1.077 ± 0.076 cd	38.232 ± 0.255 b	5.925 ± 0.304 bc	303.915 ± 20.378 b	7.847 ± 0.457 def	5.308 ± 0.280 d

Means followed by different letters in each column are significantly different (Paired bootstrap test, *p* < 0.01).

**Table 3:** Life table parameters of *Spodoptera littoralis* reared on various legume cultivars.

Legume Cultivar	*R*_0_ (Offspring per Adult)	*r* (Day^−1^)	λ (Day^−1^)	*T* (Day)
Arabi	242.34 ± 24.20 a	0.1509 ± 0.006 a	1.1628 ± 0.007 a	36.27 ± 0.54 e
Dadfar	123.59 ± 17.21 b	0.1163 ± 0.007 bc	1.1234 ± 0.008 bc	41.09 ± 0.42 bc
Ghaffar	155.58 ± 15.07 ab	0.1254 ± 0.006 bc	1.1337 ±0.006 bc	40.07 ± 0.51 d
Ofogh	180.64 ± 17.81 ab	0.1237 ± 0.005 bc	1.1316 ± 0.006 bc	41.85 ± 0.31 ab
Mashhad	118.78 ± 13.42 b	0.1115 ± 0.006 c	1.1180 ± 0.006 c	42.60 ± 0.43 a
1057	124.48 ± 28.93 b	0.1176 ± 0.006 bc	1.1248 ± 0.007 bc	40.78 ± 0.41 d
Almas	184.89 ± 22.84 ab	0.1304 ± 0.007 b	1.1393 ± 0.007 b	39.79 ± 0.35 d
Sadri	168.02 ± 15.42 ab	0.1241 ± 0.005 bc	1.1322 ± 0.005 bc	41.15 ± 0.37 bc
Yaghout	144.76 ± 16.95 ab	0.1195 ± 0.006 bc	1.1269 ± 0.007 bc	41.42 ± 0.48 bc
Dorsa	143.04 ± 15.46 ab	0.1193 ± 0.005 bc	1.1267 ± 0.006 bc	41.40 ± 0.36 bc
Saleh	112.58 ± 12.19 b	0.1140 ± 0.007 bc	1.1207 ± 0.008 bc	41.13 ± 0.29 bc

Means followed by different letters in each column are significantly different (Paired bootstrap test, *p* < 0.01). *R*_0_, net reproductive rate; *r*, intrinsic rate of increase; λ, finite rate of increase; *T*, mean generation time.

**Table 4:** Biochemical characteristics (mean ± SE) (mg/mL) of tested different legume leaves.

Legume Cultivar	Protein Content	Starch Content
Arabi	0.315 ± 0.018 abc	2.569 ± 0.028 a
Dadfar	0.223 ± 0.006 f	2.245 ± 0.049 cd
Ghaffar	0.295 ± 0.009 bcd	2.268 ± 0.010 bcd
Ofogh	0.239 ± 0.005 ef	2.247 ± 0.055 cd
Mashhad	0.304 ± 0.020 abcd	2.312 ± 0.077 abcd
1057	0.314 ± 0.003 abc	2.426 ± 0.094 abc
Almas	0.343 ± 0.010 a	2.441 ± 0.021 abc
Sadri	0.283 ± 0.005 cd	2.257 ± 0.044 bcd
Yaghout	0.225 ± 0.005 ef	2.130 ± 0.037 d
Dorsa	0.334 ± 0.005 ab	2.536 ± 0.049 ab
Saleh	0.267 ± 0.005 de	2.303 ± 0.087 abcd

Means followed by different letters in the same column are significantly different (Tukey test, *p* < 0.01).

**Table 5:** Correlation coefficients (*r*) between life table parameters or digestive enzyme activities of *Spodoptera littoralis* and biochemical traits of various legume cultivars.

Parameter	Protein Content	Starch Content
Pupal weight	−0.059 (0.742)	−0.129 (0.476)
Development time	−0.386 (0.026)	−0.325 (0.065)
Fecundity	0.082 (0.650)	0.318 (0.071)
*R*_0_	−0.026 (0.888)	−0.011 (0.954)
*r*	0.321 (0.068)	0.609 (0.047)
Amylolytic activity	0.277 (0.119)	0.619 (0.042)
Proteolytic activity	−0.041 (0.819)	−0.007 (0.968)

Correlations were evaluated based on Pearson’s correlation test (*p* < 0.01). The number in parenthesis is *p* value.

## Data Availability

The datasets generated in this study are available from the corresponding author on reasonable request.
